# In This Issue

**Published:** 2008

## WHAT IS ADDICTION?

Alcohol and other drug (AOD) use, abuse, and dependence are far-reaching phenomena. The terms substance abuse, dependence, and addiction seek to define and describe compulsive drug use; however, these terms are imprecise and must be further validated empirically. Refining these terms will help researchers and clinicians understand these phenomena more fully, which will lead to more effective diagnosis and treatment of co-morbid AOD use disorders. This introduction by Drs. Henry R. Kranzler and Ting-Kai Li reviews the articles in this issue of *Alcohol Research & Health* and describes the prevalence of AOD use disorders; the complex mechanisms behind AOD use; adverse consequences of these disorders; and challenges to the evaluation and treatment of co-occurring use, abuse, and dependence on AODs. As researchers continue to explore AOD use, these terms will continue to evolve. (pp. 93–95)

## TIMING OF ALCOHOL AND OTHER DRUG USE

The use of two or more drugs in combination at approximately the same time (i.e., simultaneous polydrug use [SPU]) is a major public health concern. When alcohol and other drugs are used together, the interaction can lead to dangerous consequences, such as overdose and even death. Dr. Christopher S. Martin discusses the functions of SPU for substance users, as well as the prevalence, patterns, and consequences of SPU. (pp. 96–99)

## AN EPIDEMIOLOGIC ANALYSIS OF CO-OCCURRING ALCOHOL AND OTHER DRUG USE AND DISORDERS

Over the past two to three decades, researchers have intensely studied the relationships between alcohol use disorders and other drug use disorders to determine how often and why these disorders co-occur. This article by Drs. Daniel Falk, Hsiao-ye Yi, and Susanne Hiller-Sturmhöfel presents epidemiologic data obtained in the National Epidemiologic Survey of Alcohol and Related Conditions (NESARC) on alcohol and other drug co-use in the general adult population of the United States, as well as in various population subgroups. These findings are providing health care policymakers and treatment planners with a comprehensive assessment of the state of the use, co-use, and co-morbidity of alcohol and other drugs and may aid researchers in developing prevention and intervention approaches. (pp. 100–110)

## THE GENETICS OF ALCOHOL AND OTHER DRUG DEPENDENCE

Studies show that certain genetic factors increase a person’s risk of both alcohol abuse and dependence and other drug abuse and dependence. Some of these factors also increase the risk of various psychiatric disorders that are characterized by uncontrolled or disinhibited behavior (i.e., externalizing disorders), including antisocial personality disorder, attention deficit/hyperactivity disorder, and conduct disorder. This article by Drs. Danielle M. Dick and Arpana Agrawal reviews and summarizes research that has identified specific genes associated with these disorders. The authors speculate that a person’s genetic risk for alcohol and other drug dependence results from both general externalizing factors and from drug use disorder–specific factors. (pp. 111–118)

## THE INFLUENCE OF STRESS ON THE TRANSITION FROM DRUG USE TO ADDICTION

Stress generally is defined as any stimulus that challenges the body’s normal physiological balance. This can include disturbances induced by alcohol and other drugs. The relationship between stress and alcohol and other drug use disorders is complex. In this article, Dr. Gary Wand reviews evidence that stress and drug use are intimately related: Stress can make a person more likely to use alcohol and other drugs, and alcohol and other drug addiction can induce stress, especially in the later stages of addiction, as drug use becomes less rewarding. Thus, a dangerous interaction between stress and drug-seeking behaviors likely exists throughout the different stages of addiction. (pp. 119–136)

## SHARED MECHANISMS OF ALCOHOL AND OTHER DRUGS

To understand alcohol and other drug addiction, researchers must first understand the changes that occur in the brain as a result of alcohol and other drug use. Although alcohol does not appear to activate specific brain chemical binding molecules, it does influence the activity of a key brain chemical (i.e., neurotransmitter) involved in addiction (γ-aminobutyric acid [GABA]), as well as endogenous opioids and cannabinoids. GABA is involved in both the immediate actions of alcohol and its long-term effects, including the development of tolerance and dependence. This article by Drs. Maureen T. Cruz, Michal Bajo, Paul Schweitzer, and Marisa Roberto reviews the current understanding of how alcohol, opioids, and cannabinoids interact with the neurotransmitter GABA and with each other. (pp. 137–147)

## DIAGNOSING CO-MORBID DRUG USE IN PATIENTS WITH ALCOHOL USE DISORDERS

Co-occurring alcohol and other drug use disorders can have serious medical and social consequences, especially when they co-occur. However, properly diagnosing alcohol and other drug use disorders can be difficult. This article by Drs. Bachaar Arnaout and Ismene L. Petrakis discusses the importance of accurately diagnosing alcohol and other drug use disorders as a first step toward treatment and recovery. It presents a step-by-step overview of how to diagnose a substance use disorder, with a special emphasis on diagnosing drug use disorders in patients who also have alcohol use disorders. (pp. 148–154)

## TREATMENT OF CO-OCCURRING ALCOHOL AND OTHER DRUG USE DISORDERS

An estimated 1.1 percent of the U.S. population has co-occurring alcohol and other drug use disorders. Relatively few studies have been conducted on the effectiveness of different treatment methods in people with co-occurring disorders; however, the evidence to date suggests that the most effective approaches are similar to those used to treat people with individual substance use disorders. This article by Drs. Albert J. Arias and Henry R. Kranzler examines how behavioral therapies such as motivation enhancement provide the “backbone” or main component of treatment for patients with alcohol and other drug use disorders and how that treatment can be supplemented by medications. The use of medications to improve outcomes in patients with co-occurring alcohol and other drug use disorders has shown initial promise, particularly for co-occurring alcohol and cocaine dependence. (pp. 155–167)

## ADOLESCENTS AT RISK FOR SUBSTANCE USE DISORDERS

Adolescents with alcohol-related problems typically also use other drugs. Accordingly, researchers are trying to identify characteristics that may predict substance use disorders in adolescents. In this article, Drs. Dawn L. Thatcher and Duncan B. Clark explain that behavioral dysregulation—a pattern of behaviors characterized by cognitive, behavioral, and emotional difficulties— can predict substance use disorders in adolescents. Other studies focus on characteristics, such as certain brain waves, that are not directly observable but which link a person’s genetic makeup to disease. This research may reveal pathways that connect genetic and early environmental influences to later substance use disorders. (pp. 168–176)

